# Mental health issues among critical care providers during the COVID-19 pandemic: the multinational PSYCOVID cross-sectional study

**DOI:** 10.1038/s41598-025-22187-9

**Published:** 2025-11-03

**Authors:** Imen Ben Saida, Salma Raouene, Radhouane Toumi, Emna Ennouri, Salma Jerbi, Khaoula Meddeb, Maher Maoua, Mohamed Boussarsar

**Affiliations:** 1 Research Laboratory LR12SP09 Heart Failure, Farhat Hached University Hospital, University of Sousse, Sousse, Tunisia; 2 Medical Intensive Care Unit, Faculty of Medicine of Sousse. , Farhat Hached University Hospital, Sousse, Tunisia; 3 Occupational Medicine Department, Faculty of Medicine of Sousse, Sahloul University Hospital, University of Sousse, Sousse, Tunisia

**Keywords:** COVID-19, Post-traumatic stress disorder, Anxiety, Depression, Burnout, Mental health, Healthcare worker, Critical care, Psychology, Health care, Health occupations

## Abstract

The novel coronavirus disease 2019 (COVID-19) outbreak has presented a challenging situation for frontline healthcare workers. This study aimed to assess the frequency and severity of mental health issues among critical care providers (CCPs) during the early phase of the pandemic and to determine associated factors. It was a cross-sectional study conducted from May 2020 to August 2020. A self-reported questionnaire was used to collect CCPs characteristics and psychological impact as evaluated by three validated inventory scales: the Copenhagen Burnout Inventory (CBI), Depression, Anxiety and Stress Scale-21 (DASS-21), and Impact of Event Scale-Revised (IES-R). Among 244 participants, fear was prevalent, involving risk of infection 79(32.4%), family exposure 201(82.4%), lack of PPEs 86(35.2%), increased workload and insufficient social support 101(41.4%), lack of sufficient communication 99(40.6%), and stigmatization 63(25.8%). High prevalence rates were observed for burnout (personal 54.1%, work-related 55.3%, patient-related 41.8%), depression (67.2%), anxiety (63.1%), and stress (58.6%). Additionally, 27% met criteria for probable PTSD. Multivariate analysis identified significant associations between female sex, nursing profession, and working in a low- and middle-income country with various mental health outcomes. Understanding the psychological challenges faced by these providers can inform the development of more effective preparedness and response strategies for future pandemics.

**Supplementary Information:**

The online version contains supplementary material available at 10.1038/s41598-025-22187-9.

## Introduction

Severe Acute Respiratory Syndrome Coronavirus 2 (SARS-CoV-2) is a new virus initially identified in Wuhan, Hubei Province, China, in December 2019^1^. The World Health Organization (WHO) declared the 2019 Coronavirus disease (COVID-19) outbreak as a global pandemic with serious consequences in March 2020^1^. This unprecedented and prolonged crisis affects frontline healthcare workers (HCWs), particularly those in emergency departments, infection control units, intensive care units, and COVID-19 patient wards^2^. Previous studies have indicated that mental health disorders among HCWs are common after pandemics^3^. Indeed, pandemics, including severe acute respiratory syndrome (SARS), the Middle East respiratory syndrome (MERS), and influenza A virus subtype H1N1 (H1N1), have had adverse mental health effects on healthcare providers^3^. Dealing with infected patients and facing the immediate challenges of combating a highly contagious infection can lead to painful experiences of fear, anxiety, and even social prejudice and stigma, exposing them to an increased risk of developing adverse health issues, including depression, anxiety disorders, substance abuse, increased suicidal behavior, and post-traumatic stress disorder (PTSD)^4–6^. Mental health issues among HCWs are prevalent but often overlooked public health concerns prior to the advent of COVID-19. In regular circumstances, critical care providers (CCPs) are required to continuously acquire new skills, endure extended working hours, and confront mortality routinely^7^. A considerable portion might have already been on the brink of burnout^8^. The COVID-19 outbreak has likely intensified the emotional strain, stemming from concerns about infection, issues related to preparedness, the availability of Personal Protection Equipment (PPE), diagnostic and therapeutic uncertainties inherent in an emerging disease, and the severity of patients, leading to elevated workloads^9^. Additionally, the disorganization, dehumanization, and social distancing measures (government-imposed lockdowns or stay-at-home orders) may further contribute to heightened stress levels^10,11^.

Identifying modifiable and non-modifiable predictors of CCPs mental health in the COVID-19 outbreak may inform targeted interventions to improve provider experiences in future pandemics.

This study aimed to comprehensively examine the multifaceted mental health challenges faced by CCPs in both high-income and low- and middle-income countries (LMICs) countries. Specifically, the study aimed to assess the frequency and severity of mental health issues among CCPs during the COVID-19 pandemic and to identify associated factors.

## Methods

### Study design and participants

For this multinational study, an online cross-sectional survey was conducted from May 2020 to August 2020, during the first wave of the worldwide COVID-19 pandemic. Potential respondents from departments that had previously collaborated with members of the study steering committee (MB and IB in the authors’ list) were invited to participate. The steering committee targeted 13 sites from three countries for inclusion in the study. Departments were eligible if they were providing care for critically ill COVID-19 patients. All CCPs working in those departments were considered eligible participants for the study. A formal sample size calculation was not performed. Instead, our pragmatic approach aimed for a comprehensive representation of this specific population by including all CCPs who met the inclusion criteria and consented during the study period.

All participants provided electronic consent before accessing the questionnaire. Those who agreed were guided to the survey, and those who declined were automatically exited. Responding to all questions was mandatory for participants to successfully submit their surveys. To encourage participation, personalized feedback was immediately emailed to each responder, providing insights into their mental health status. The questionnaire was distributed to potential respondents via Google Forms, and they were encouraged to share it within their networks in Tunisia, Morocco, and France. The Ethics and Research Committee of Farhat Hached University Hospital, Sousse, Tunisia (IORG 0007439 ERC03092023), granted an exemption for this study, which explicitly extended to participants in Morocco and France. All participants provided informed consent.

### Collected data

A self-reported questionnaire was used to collect CCPs characteristics, factors that may interfere with psychological outcomes, and psychological screening scales. The questionnaire was developed by the study steering committee, written in French, using simple and understandable vocabulary. The study’s title, framework, and objectives were explicitly outlined in the questionnaire. The questionnaire consisted of three parts: Demographic information, and factors that may interfere with psychological outcomes followed by three validated screen scales.

- Demographic information included: Age (years), gender, environment (urban, rural), and country of origin (Tunisia, Morocco, or France).
- Factors that may interfere with psychological outcomes^10–13^: habitat during the pandemic (alone, with family/roommate, moved during the pandemic), workplace (intensive care unit, emergency department, adult inpatient ward), professional title (nurse, junior doctor, senior doctor), professional experience (years), weekly working hours, exclusive night work, fear (yes or no), and cause of fear (the fear of being infected, the fear of exposure to family members, lack of access to PPEs, lack of sufficient communication and updated information, increased workload and lack of social support, stigmatization).
- Psychological internationally validated screening scales were used to identify potential mental disorders among HCWs: the Copenhagen Burnout Inventory (CBI), Depression, Anxiety and Stress Scale-21 (DASS-21), and Impact of Event Scale-Revised (IES-R).

The questionnaire was electronically distributed via Google Forms to relevant departments participating in the study. https://docs.google.com/forms/d/e/1FAIpQLSe14TBvT_7t99zQIkkDQL4z1scgEn4GQSJWUctEd4utGy6Msw/viewform?usp=sf_link.

To encourage responders to complete the questionnaire, personalized feedback was promptly e-mailed to the responder.

### Applied definitions

The psychological impact was assessed using the French version of three validated inventory scales (CBI, DASS-21, and IES-R).

- The CBI is a 19-item self-report questionnaire specifically designed to evaluate various dimensions of burnout on a five-point Likert scale, ranging from 0 to 4, where 0 means “never " and 4 means “always “. This inventory consists of three subscales: personal burnout (6 items: questions 1–6), work-related burnout (7 items: questions 7–13), and client-related burnout (6 items: questions 14–19). Scores ranging from 50 to 74 are categorized as “moderate”, scores between 75 and 99 are classified as “high”, and a score of 100 is deemed “severe” burnout. It has been widely used to assess diverse categories of HCWs across multiple countries and has demonstrated strong psychometric properties for measuring occupational burnout^14,15^.
- The DASS-21 is a 21-item self-report assessment tool used to measure the severity of symptoms on a four-point scale. Each response is scored from 0 to 3, where 0 means “Does not apply to me at all” and 3 means “Applies to me very much or most of the time”. It consists of three subscales, each with 7 items, evaluating depression (items 3, 5, 10, 13, 16, 17, and 21), anxiety (items 2, 4, 7, 9, 15, 19, and 20), and stress (items 1, 6, 8, 11, 12, 14, and 18).

Cut-off scores for depression, anxiety, and stress are > 9, > 7, and > 14, respectively. In the DASS-21 depression subscale, scores of 10–13 are classified as “mild,” 14–20 as “moderate,” 21–27 as “severe,” and 28–42 as “extremely severe” depression. For the DASS-21 anxiety subscale, scores are categorized as “mild”^8,9^, “moderate”^10^, “severe”^13–17^, and “extremely severe”^18–41^. The DASS-21 stress subscale score is assessed as “mild”^13–16^, “moderate”^17–23^, “severe”^24–31^, and “extremely severe” stress^32–40^. The original internal consistency coefficients for this scale are adequate, both for the overall scale (α = 0.93) and for the subscales (depression α = 0.88, anxiety α = 0.82, stress α = 0.90)^17,18^.

The IES-R is a self-report questionnaire designed to evaluate subjective distress on a 5-point scale, ranging from 0 (not at all) to 4 (extremely) and provide a preliminary diagnosis of PTSD, emphasizing three response categories: (i) intrusion subscale (items 1, 2, 3, 6, 9, 14, 16, 20), (ii) avoidance subscale (items 5, 7, 8, 11, 12, 13, 17, 22), and (iii) hyperarousal subscale (items 4, 10, 15, 18, 19, 21). The IES-R is scored with a total ranging from 0 to 88 and three sub-scores representing intrusion (0–32), avoidance (0–32), and hyperarousal (0–24). A diagnosis of PTSD was made when the total IES-R score exceeded 33^19,20^.

### Statistical analysis

Statistical analyses were conducted using SPSS version 24.0 [IBM, US]. The Kolmogorov-Smirnov test was employed to analyze the distributions of variables. Continuous variables were presented as either the mean ± standard deviation (SD) or as the median and interquartile range. A significance threshold of *P* < 0.05 was applied. The comparison between two groups utilized independent samples t-tests for normally distributed variables and the Mann-Whitney test for non-normally distributed quantitative variables. Pearson’s chi-squared test and Fisher’s test were employed for categorical variables. Correlations between CBI, DASS subscales, and IES-R were assessed using Pearson or Spearman if the data were normally or non-normally distributed. Spearman’s correlation coefficients. The correlation coefficient (R) was considered high when it was > 0.70, good when it was between 0.50 and 0.70, fair between 0.30 and 0.50, and weak or no association if it was < 0.30. Logistic regression analysis was performed to identify variables independently and significantly associated with CBI subscales (score ≥ 50), DASS depression (score > 9), DASS 21 anxiety (score > 7), DASS stress (score > 14) and IES-R (score ≥ 33). Variables with a p-value less than 0.05 in the univariate analyses were considered for inclusion in the multivariable logistic regression model.

## Results

**Fig. 1:**
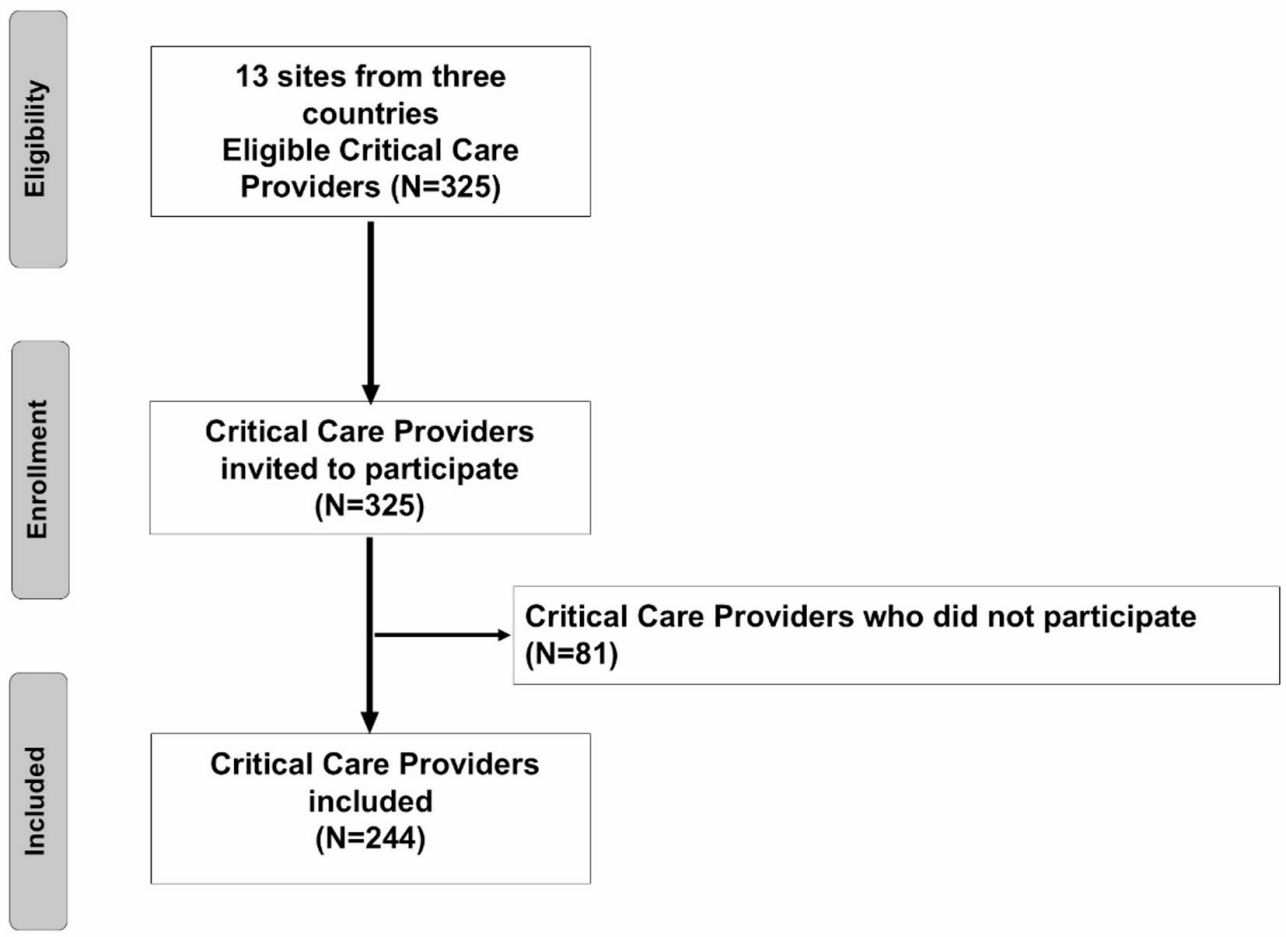
Flow diagram of participants.

Two hundred forty-four CCPs out of 325 completed the surveys (response rate 75.1%). The study flow diagram is displayed in Fig. 1. All key variables and outcomes in the present study were complete, with no missing data.

### Participants’ characteristics

One hundred sixty-one responses were from Tunisia, 70 from France, and 13 from Morocco. Responders’ characteristics are summarized in Table 1.

**Table 1:** Participants’ demographic characteristics and factors that may interfere with psychological outcomes.

	*N* = 244
Age (years) median[IQR]	30[27–37]
Female n(%)	155(63.5)
Urban environment n(%)	191(78.3)
Country of origin n(%)	
Tunisian	161(66)
Moroccan	13(5.3)
French	70(28.7)
Habitat during pandemic n(%)	
Alone	56(23)
With family/roommate	151(61.9)
Moved during pandemic	37(15.1)
Workplace n(%)	
Intensive care unit	194(79.5)
Emergency department	32(13.1)
Adult inpatient ward	18(7.4)
Professional title n(%)	
Senior Doctor	62(25.4)
Junior Doctor	99(40.6)
Nurse	83(34)
Professional experience years median[IQR]	4[2–9]
Weekly working hours median[IQR]	48[36–59.5.5]
Exclusive night work n(%)	88(36.1)

The cohort primarily comprised 161(66%) physicians and 83(34%) nurses. This group was predominantly female (63.5%), with a median professional experience of 4 years. 78.3% of the participants were from urban environments, reflecting the primary settings for COVID-19 critical care

### Psychological impact on CCPs

#### Fear

All participants experienced fear. The causes of fear are displayed in Table 2.

**Table 2:** Prevalence of specific factors contributing to fear among critical care providers.

	*N* = 244
The fear of being infected	79(32.4)
The fear of exposure to family members	201(82.4)
Lack of access to PPEs	86(35.2)
Lack of sufficient communication and updated information	99(40.6)
Increased workload and lack of social support	101(41.4)
Stigmatization	63(25.8)

All variables are displayed as n (%). PPEs: Personal protection equipment. Fear of familial transmission was a predominant concern among CCPs, exceeding anxieties related to personal infection or resource scarcity

#### Burnout

**Fig. 2:**
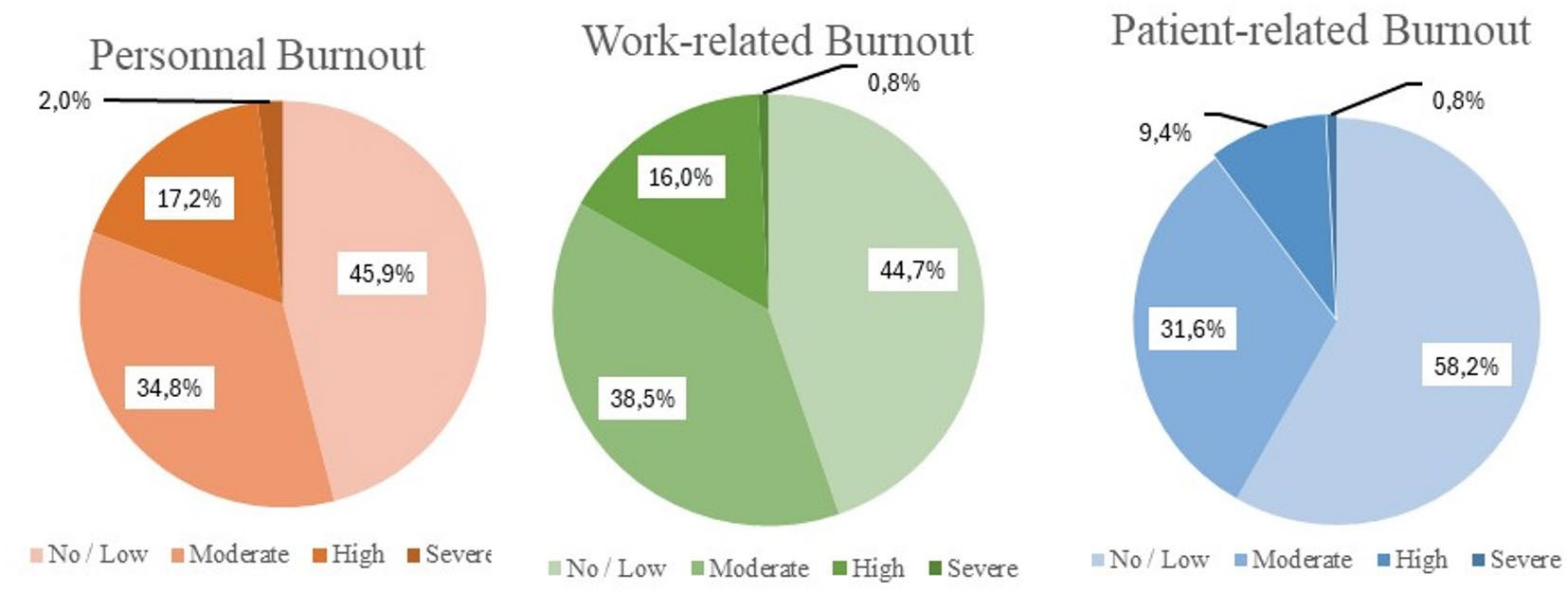
Percentage of participants experiencing Burnout stratified by severity using the Copenhagen Burnout Inventory subscales (*N* = 244).

The median CBI subscale scores were: personal burnout 60[40–80]; work-related burnout 72.7[50–95], and client-related burnout 50[30–73.75.75]. One hundred thirty-two (54.1%) participants reported personal burnout, 135(55.3%) reported work-related burnout, and 102(41.8%) reported patient‐related burnout. Figure 2 illustrates the severity of burnout as measured by the Copenhagen Burnout Inventory subscales. These high percentages across all burnout subscales underscore the significant emotional and physical exhaustion experienced by CCPs, particularly concerning their work-related responsibilities during the early pandemic phase.

#### Depression, anxiety, and stress

**Fig. 3:**
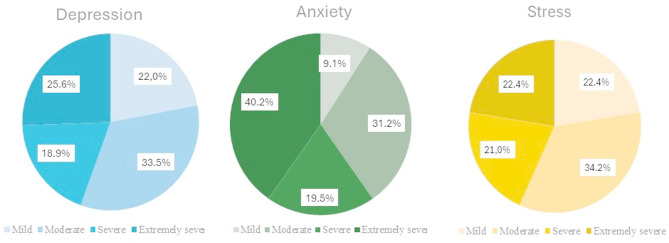
Percentage of participants experiencing adverse psychological impact, stratified by severity using DASS-21 (depression: *n* = 164;).

Based on the DASS-21 scores, 164(67.2%), 154(63.1%), and 143(58.6%) of participants had respectively depression, anxiety, and stress. Among the 244 participants, rates of moderate to extremely severe depression, anxiety, and stress were 52.4%, 57.4%, and 45.5%, respectively. Figure 3 displays the percentage of participants experiencing adverse psychological effects, stratified by severity using the DASS-21 scales within the affected subgroups.

The notably high rates of moderate to extremely severe symptoms across all three DASS-21 subscales highlight a widespread and clinically significant mental health burden within the CCPs cohort during the study period.

#### PTSD

The overall median IES-R score for all participants was 20, with a range of 9–33.

The most frequent symptoms of subjective trauma distress sub-scale were median (range): intrusion, 7^4–12^ and avoidance, 7^3–12^ and hyperarousal, 5^2–10^. 27% (*n* = 66) of them reported symptoms of ‘probable PTSD’ according to a cut-off score of 33 on the IES-R.

#### Correlations of mental health outcomes

All burnout subscales were significantly correlated with depression, anxiety, and stress scales, and with IES-R total and its subscales. A fair and good association was identified between depression, anxiety, and stress scales and IES-R total and its subscales (Table 3).

**Table 3:** Spearman’s rho correlations between.

	DASS depression	DASS anxiety	DASS stress	IES-*R* total	Intrusion	Avoidance	Hyperarousal
CBI personal	0.605^a^	0.51^a^	0.647 ^a^	0.585^a^	0.547^a^	0.523^a^	0.603^a^
CBI work-related	0.566^a^	0.418^a^	0.604^a^	0.564^a^	0.529^a^	0.505^a^	0.569^a^
CBI client-related	0.491^a^	0.410^a^	0.548^a^	0.591^a^	0.560^a^	0.544^a^	0.580^a^
DASS depression	-	-	-	0.523^a^	0.462^a^	0.513^a^	0.520^a^
DASS anxiety	-	-	-	0.483^a^	0.429^a^	0.477^a^	0.508^a^
DASS stress	-	-	-	0.601^a^	0.527^a^	0.531^a^	0.637^a^

^a^Correlation is significant at the 0.01 level (2-tailed); CBI Copenhagen burnout inventory; DASS-21 Depression, anxiety and stress scale; Impact of event scale revised.

### Factors associated with adverse mental health problems

Univariate and multivariate analyses revealed significant associations between female sex, nurse profession, working in a LMICs, and various mental health outcomes (Tables 4, 5 and 6). These consistent associations underscore that female CCPs, nurses, and those working in LMICs are particularly vulnerable to adverse psychological outcomes.

**Table 4:** Univariate analysis of factors associated with Burnout.

Variables	Personal burnout			Work-related burnout			Client-related burnout			
	No (*N* = 112)	Yes (*N* = 132)	*P*	No (*N* = 109)	Yes (*N* = 135)	*P*	No (*N* = 142)	Yes (*N* = 102)	*P*	
Age (years) median[IQR]	31[27–38]	30[27–36.75.75]	0.583	31[27–37]	30[27–36]	0.546	30[27–37]	30.5[27–37]	0.796	
Female sex n(%)	60(53.6)	95(72)	0.03	63(57.8)	92(68.1)	0.095	81(57)	74(72.5)	0.013	
Urban environment n(%)	90(80.4)	101(76.5)	0.468	88(80.7)	103(76.3)	0.403	115(81)	76(74.5)	0.226	
Habitat during pandemic n(%) Alone With family/roommate Moved during pandemic	27(24.1) 70(62.5) 15(13.4)	29(22) 81(61.4) 22(16.7)	0.755	25(22.9) 69(63.3) 15(13.8)	31(23) 82(60.7) 22(16.3)	0.852	33(23.2) 93(65.5) 16(11.3)	33(22.5) 58(56.9) 21(20.6)	0.127	
Workplace n(%)										
Intensive care unit	92(82.1)	102(77.3)		89(81.7)	105(77.8)		112(78.9)	82(80.4)		
Emergency department	13(11.6)	19(14.4)	0.639	11(10.1)	21(15.6)	0.429	17(12)	15(14.7)	0.404	
Adult inpatient ward	7(6.3)	11(8.3)		9(8.3)	9(6.7)		13(9.2)	5(4.9)		
Nurse	32(28.6)	51(38.6)	0.098	29(26.6)	54(40)	0.028	38(26.8)	45(44.1)	0.005	
Professional experience years median[IQR]	4[2–10]	4[2–8]	0.289	4[2–10]	4[2–8]	0.602	4[2–10]	4[2–8]	0.866	
Weekly working hours median[IQR]	48[36–60]	48[38–55]	0.785	48[36–60]	48[38–55]	0.559	48[36–60]	48[38.75–58.5]	0.99	
Dealing with critically ill patients n(%)	105(93.8)	121(91.7)	0.535	100(91.7)	126(93.3)	0.637	129(90.8)	97(95.1)	0.210	
Direct contact with COVID-19 patients n(%)	108(96.4)	123(93.9)	0.363	103(94.5)	128(95.5)	0.713	132(93.6)	99(97.1)	0.222	
Exclusive night work n(%)	37(33)	51(38.6)	0.364	33(30.3)	55(40.7)	0.091	47(33.1)	41(40.2)	0.255	
Working in a LMICs	70(62.5)	104(78.8)	0.005	69(63.3)	105(77.8)	0.013	94(66.2)	80(78.4)	0.003	

P: comparison between groups (Mann-Whitney test for continuous variables and chi-square (χ2) test for categorical ones)

**Table 5:** Univariate analysis of factors associated with depression, anxiety, stress and PTSD.

Variables	Depression			Anxiety			Stress			PTSD		
	No (*N* = 80)	Yes (*N* = 164)	*P*	No (*N* = 90)	Yes (*N* = 154)	*P*	No (*N* = 101)	Yes (*N* = 143)	*P*	No (*N* = 178)	Yes (*N* = 66)	*p*
Age (years) median[IQR]	31[27–40]	30[27–36]	0.34	30.5[26.75–38.25]	30[27–36]	0.98	30[27–38.5.5]	30[27–36]	0.515	30[27–37]	30[27–35.25.25]	0.475
Female sex n(%)	41(51.2)	114(69.5)	0.005	43(47.8)	112(72.7)	0.000	52(51.5)	103(72)	0.001	105(59)	50(75.8)	0.016
Urban environment n(%)	64(80)	127(77.4)	0.649	74(82.2)	117(76)	0.253	81(80.2)	110(76.9)	0.541	137(77)	54(81.8)	0.414
Habitat during pandemic n(%)												
Alone	20(25)	36(22)		17(18.9)	39(25.3)		21(20.8)	35(24.5)		41(23)	15(22.7)	
With family/roommate	50(62.5)	101(61.6)	0.676	64(71.1)	87(56.5)	0.064	66(65.3)	85(59.4)	0.645	113(63.5)	38(57.6)	0.472
Moved during pandemic	10(12.5)	27(16.5)		9(10)	28(18.2)		14(13.9)	23(16.1)		24(13.5)	13(19.7)	
Workplace n(%)												
Intensive care unit	67(83.8)	127(77.4)		73(81.1)	121(78.6)		80(79.2)	114(79.7)		141(79.2)	53(80.3)	
Emergency department	9(11.3)	23(14)	0.473	9(10)	23(14.9)	0.462	12(11.9)	20(14)	0.687	22(12.4)	10(15.2)	0.528
Adult inpatient ward	4(5)	14(8.5)		8(8.9)	10(6.5)		9(8.9)	9(6.3)		15(8.4)	3(4.5)	
Nurse n(%)	24(30)	59(36)	0.355	29(32.3)	54(35.1)	0.651	29(28.7)	54(37.8)	0.142	54(30.3)	29(43.9)	0.046
Professional experience years median[IQR]	4[2–10]	4[2–8]	0.638	4[2–10]	4[2–8]	0.348	4[2–10]	4[2–8]	0.226	4[2–10]	4[2–7.25.25]	0.358
Weekly working hours median[IQR]	48[36–60]	48[36–55.75.75]	0.321	48[36–60]	48[36–56]	0.810	48[36–60]	48[37–55]	0.806	48[36–60]	48[39–56.5.5]	0.507
Dealing with critically ill patients n(%)	76(95)	150(91.5)	0.321	82(91.1)	144(93.5)	0.49	92(91.1)	134(93.7)	0.441	163(91.6)	63(95.5)	0.303
Direct contact with COVID-19 patients n(%)	77(96.3)	154(94.5)	0.549	86(95.6)	145(94.8)	0.785	98(97)	133(93.7)	0.232	167(94.4)	64(97)	0.402
Exclusive night work n(%)	25(31.3)	63(38.4)	0.274	32(35.6)	56(36.4)	0.899	31(30.7)	57(39.9)	0.142	63(35.4)	25(37.9)	0.719
Working in LMICs	44(55)	126(76.8)	0.000	44(48.9)	126(81.8)	0.000	64(63.4)	106(74.1)	0.072	120(67.4)	50(75.8)	0.208

P: comparison between groups (Mann-Whitney test for continuous variables and chi-square (χ2) test for categorical ones)

**Table 6:** Multivariate analysis of factors associated with mental health outcomes.

	Variables	OR	95%IC	*P*
Personal burnout	Female sex	2.09	[1.218–3.584]	0.007
	Working in LMICs	2.07	[1.166–3.69]	0.013
Work-related burnout	Working in LMICs	2.586	[1.412–4.736]	0.002
	Nurse	2.361	[1.309–4.259]	0.004
Client-related burnout	Female sex	1.849	[1.048–3.262]	0.034
	Working in LMICs	2.365	[1.237–4.519]	0.009
	Nurse	2.692	[1.496–4.843]	0.001
Depression	Female sex	1.996	[1.135–3.513]	0.016
	Working in LMICs	2.688	[1.494–4.836]	0.001
Anxiety	Female sex	2.744	[1.541–4.886]	0.001
	Working in LMICs	4.498	[2.452–8.254]	0.000
Stress	Female sex	2.426	[1.412–4.141]	0.001
PTSD	Female sex	2.173	[1.149–4.109]	0.017

## Discussion

This cross-sectional study reveals a substantial psychological toll on CCPs during the early phase of the COVID-19 pandemic. High rates of burnout, depression, anxiety, stress, and probable PTSD were observed. Fear was prevalent, primarily driven by concerns about infection, family exposure, lack of PPEs, insufficient social support, increased workload, lack of sufficient communication, and stigmatization. Notably, female providers, nurses, and those working in LMICs were more vulnerable to adverse mental health outcomes.

This study possesses several strengths: First, the study included a large sample of CCPs. Second, internationally recognized and validated screening scales (CBI, DASS-21 and IES-R) were used to measure burnout, depression, anxiety, stress, and PTSD. Third, the study investigated a comprehensive range of mental health outcomes. This multifaceted approach provides a more complete picture of the psychological impact of the pandemic on CCPs. Fourth, the study included CCPs from multiple centers, including those in the Maghreb region (Tunisia, Morocco), a low-middle-income region, and a developed country (France). This broadens the generalizability of the findings. Finally, the immediate feedback provided to participants may enhance their understanding of their own mental health status.

Consistent with previous studies^11,20–22^, CCPs reported experiencing a wide range of stressors, including personal and familial risk (fear of personal infection and infecting family members), inadequate resources (concerns about inadequate PPEs, shortage of beds), increased workload, insufficient communication and stigmatization.

Previous studies on COVID-19 examining psychological symptoms among frontline HCWs during the first wave of the pandemic have generally reported lower pooled prevalence rates than those found in the current research^23,24^. A systematic review and meta-analysis of mental health status of HCWs during the COVID-19 pandemic by Huang et al^25^. found 47% of HCWs reported job burnout, 38% experienced anxiety, 34% reported depression, 30% had acute stress disorder, and 26% had PTSD. This discrepancy may be attributed to the fact that the present study participants’ were directly treating critically ill COVID-19 patients, which appears to be an independent risk factor for all psychiatric symptoms^26^.

The present study confirms findings from various studies^10,20,27^ reporting a strong psychological impact of COVID-19 on female nurses working in LMICs.

As a cornerstone of caring for critically ill patients, nurses experiencing burnout may be less effective in providing high-quality care, potentially leading to worse patient outcomes.

Nurses’ increased exposure to COVID-19 patients, due to more frequent contact with high-risk individuals, could contribute to their higher psychological harm^27^.

Studies^2,20,24,28–30^ have consistently shown that nurses were vulnerable population and there is a strong association between inadequate nurse staffing and higher rates of burnout. Burnout can lead to a range of mental health problems, including depression, anxiety, and PTSD. Given the complexity of caring for patients with COVID-19 in critical care units, it is crucial to establish a nurse-to-patient ratio.

Baraka et al.^31^ demonstrated that the unavailability of hospital resources and the number of colleagues infected with COVID-19 were the strongest predictors of stress, anxiety, and depression among nurses.

Doctors may also have been more likely to underreport symptoms compared to nurses, although this possibility could not be explored further within the scope of the present study^32,33^. In the systematic review and meta-analysis by Huang et al.^25^, there was no evidence of differential prevalence of mental health disorders between doctors and nurses.

Hammami et al.^11^ found that female sex was associated with anxiety and depression. Women are more likely to experience psychosocial risks than men are. Additionally, the pressure to balance work and family responsibilities can contribute to increased stress and exhaustion among women^6^.

Azoulay et al.^34^ found personal perceptions, such as fear, to be significant factors of the psychological burden of the COVID-19 pandemic among French HCWs. This relationship could not be determined in the present study given the widespread experience of fear among all participants in our study population.

LMICs in the Maghreb region (Algeria, Morocco, Tunisia, and Libya) faced unique challenges during the COVID-19 pandemic, which likely contributed to the heightened mental health struggles of HCWs. The limited resources, inadequate staffing in low-income ICUs and emergency departments, and the lack of necessary psychological and logistical resources to effectively cope with the rapidly escalating pandemic and economic crisis may have contributed to the higher rates of mental health problems observed among Maghrebian CCPs in the present study^11^. A worldwide survey^35^ conducted in ICUs across different regions (Europe, America, Asia, and Africa) demonstrated that female CCPs and nurses were more likely to experience burnout than their male counterparts. Insufficient resources were reported to be associated with burnout in all regions.

The estimated PTSD rate in the present study was 27%. A higher rate was reported in the study of Greenberg et al.^20^ in front-line UK staff (40%) which was around nine times that found within the general population. In fact, the COVID-19 pandemic has heightened the risk of PTSD worldwide among front line HCWs due to increased patient mortality and the threat of personal injury^36^.

The present study revealed correlations between burnout, depression, anxiety, and stress scales, which may partly reflect the inherent overlap among these mental health constructs.

There are some potential limitations in the present study. First, the study relied on self-reported questionnaires to assess mental health outcomes. Participants may have over- or under-reported mental health symptoms. Second, given the voluntary nature of the survey, CCPs who were particularly impacted emotionally may have been more likely to participate. Third, the imbalanced distribution of participating Maghrebian countries, with Tunisia being overrepresented, limits the generalizability of our findings across the entire Maghrebian region. Fourth, with nurses constituting only 34% of our sample, the generalizability and external validity of subgroup findings concerning this profession are limited. Fifth, data collection occurred solely during the first wave of COVID-19 cases, which may limit generalizability to later pandemic stages. Finally, the pandemic’s psychological impact on CCPs may have evolved over subsequent waves.

Despite these limitations, the findings from this study offer valuable insights for planning and responding to future pandemics to improve the psychological well-being of CCPs and, thus their security and patient management quality.

Female nurses in LMICs, particularly in the Mediterranean region, face numerous challenges that significantly affect their mental health. These challenges include the devalued status of the nursing profession, often perceived as subordinate to physicians^37^. In low-income settings, this professional devaluation is amplified by chronic underfunding of healthcare systems, leading to persistent shortages of essential resources, including personnel, equipment, and basic medical supplies^31^. This scarcity directly contributes to excessive workloads, extended duty hours, and frequent ethical dilemmas, all of which are significant occupational stressors^38^. Societal expectations and traditional gender roles place a heavy burden on female nurses, who often juggle demanding careers with significant domestic responsibilities^39^. Moreover, the COVID-19 pandemic has exacerbated these challenges, with increased workloads, inadequate resources, and heightened fears of infection contributing to high levels of burnout, anxiety, and depression among female nurses in these resource-limited settings^40–42^. Addressing these complex issues requires a multi-pronged approach that includes improving the social and economic status of the nursing profession, promoting gender equity, and providing adequate support systems for the mental health and well-being of female nurses^43^.

The findings of this study highlight the urgent need for targeted interventions to mitigate the psychological toll on CCPs during future health crises. We recommend that healthcare institutions prioritize establishing accessible and confidential mental health support, such as psychological debriefing sessions, individual counseling, group therapy, and peer support programs. They should also optimize staffing to prevent burnout by ensuring adequate rest periods and implementing flexible scheduling, and enhancing transparent communication. Policymakers and governments must strengthen PPE supply chains, integrate comprehensive psychosocial preparedness into national emergency plans, and invest in healthcare infrastructure and workforce equity, especially in LMICs. For the research community, future efforts should focus on longitudinal studies to track long-term impacts and develop context-specific interventions that consider cultural and resource limitations, ultimately ensuring a robust and resilient critical care workforce.

## Conclusion

This multinational cross-sectional study revealed a significant and multifaceted psychological toll on CCPs during the early phase of the COVID-19 pandemic. Based on CBI results, a high prevalence of burnout was observed, significantly impacting personal (54.1%), work-related (55.3%), and patient-related (41.8%) domains. Furthermore, substantial rates of mental health symptoms were identified using the DASS-21, including depression (67.2%), anxiety (63.1%), and stress (58.6%). Additionally, 27% of participants met criteria for probable PTSD as measured by the IES-R. Female sex, the nursing profession, and working in LMICs were significantly associated with various adverse mental health outcomes. Understanding these specific psychological challenges and associated risk factors is crucial for informing the development of more effective preparedness and response strategies for future pandemics by relevant stakeholders.

## Supplementary Information

Below is the link to the electronic supplementary material.

Supplementary Material 1

## Data Availability

The datasets used and/or analyzed during the current study are available from the corresponding author upon reasonable request.

## Abbreviations

CCPs: Critical care providers
CBI: Copenhagen burnout inventory
DASS-21: Depression, anxiety and stress scale-21
IES-R: Impact of event scale-revised
WHO: World health organization
COVID-19: Coronavirus disease 2019
SARS-CoV-2: Severe acute respiratory syndrome coronavirus 2
HCWs: Healthcare workers
PTSD: Post-traumatic stress disorder
PPE: Personal protection equipment
